# A *Trichinella spiralis* serine protease triggers gut epithelial apoptosis and destroys the barrier integrity to mediate larval invasion

**DOI:** 10.1371/journal.pntd.0013680

**Published:** 2025-10-30

**Authors:** Qi Qi Lu, Xin Zhuo Zhang, Pei Kun Cong, Jin Yi Wu, Tian Ran Gao, Shao Rong Long, Ruo Dan Liu, Zhong Quan Wang, Jing Cui

**Affiliations:** Department of Parasitology, School of Basic Medical Sciences, Zhengzhou University, Zhengzhou, China; Zhejiang Wanli University, CHINA

## Abstract

**Background:**

Larval invasion of gut mucosa is a crucial procedure in *Trichinella spiralis* infection. Previous studies showed that *T. spiralis* excretory-secretory proteins (ESP) disrupted gut epithelial integrity and promoted larval invasion by inducing apoptosis. However, the key molecules of the ESPs involved in this process are unknown. A serine protease (TsSPc) in *T. spiralis* ESPs was identified to be capable of promoting larval invasion. The aim of this study was to investigate whether TsSPc induces gut epithelial apoptosis, disrupts the barrier function, promotes larval invasion, and elucidates its acting mechanism.

**Methodology/principal findings:**

The results of DAPI, TUNEL and Annexin V/PI staining, immunofluorescence test (IFT) and flow cytometry showed that rTsSPc triggered Caco-2 cell apoptosis. The results of qPCR and Western blot revealed that rTsSPc significantly up-regulated the expression of Caco-2 cell apoptosis-related proteins (Caspase 3, Caspase 9 and Caspase 8, Bax and Cytochrome c), activated the apoptosis pathway, and thereby induced Caco-2 apoptosis. rTsSPc bound and interacted with PGAM5 receptor in Caco-2 cells and induced apoptosis, reduced the tight junctions (TJs) expression levels and damaged the Caco-2 monolayer integrity and barrier function, and promoted larval invasion. When gut epithelial PGAM5 receptor and apoptosis were inhibited by PGAM5-specific siRNA, inhibitor (LFHP-1c) and apoptosis inhibitor (Z-VAD-FMK), trans-epithelial electrical resistance (TEER) and TJs expression were obviously increased, and intestinal permeability was evidently decreased. Additionally, larval invasion of Caco-2 monolayer was also decreased by siPGAM5 and inhibitor pretreatment. These findings indicated that inhibition of PGAM5 receptor and apoptosis prevented rTsSPc from damaging gut epithelial integrity and larval invasion.

**Conclusions:**

rTsSPc binding and interacting with PGAM5 triggered gut epithelial apoptosis, reduced the TJs expression and damaged gut barrier function, thereby mediated larval invasion. TsSPc might be a candidate vaccine target to interdict larval invasion and *T. spiralis* infection.

## Author summary

Previous studies showed that *Trichinella spiralis* excretory-secretory proteins (ESP) disrupted intestinal epithelial barrier and promoted larval invasion by inducing apoptosis. However, the key molecules of the ESPs involved in this process are unknown. A *T. spiralis* serine protease (TsSPc) as an important component of ESPs has been confirmed to disrupt intestinal epithelial integrity. The aim of this study was to investigate whether TsSPc induces intestinal epithelial apoptosis, disrupts the barrier function, promotes larval invasion, and elucidates its acting mechanism. The results showed that rTsSPc damaged intestinal epithelium and induced enterocyte apoptosis. rTsSPc interacted with PGAM5 and induced apoptosis, reduced the tight junctions (TJs) expression levels and damaged the Caco-2 monolayer integrity barrier and promoted larval invasion. When PGAM5 and apoptosis were inhibited by PGAM5-specific siRNA, inhibitor (LFHP-1c) and apoptosis inhibitor (Z-VAD-FMK), the trans-epithelial electrical resistance (TEER) and TJs expression were obviously increased and intestinal permeability was evidently decreased. Additionally, larval invasion of Caco-2 monolayer was also decreased by siPGAM5 and inhibitor pretreatment. These results indicated that inhibition of PGAM5 and apoptosis prevented rTsSPc from damaging intestinal epithelial integrity and larval invasion. rTsSPc binding and interacting with PGAM5 induced gut epithelial apoptosis, reduced the TJs expression and damaged gut barrier function, thereby mediated larval invasion. TsSPc may serve as a candidate molecular target to block *T. spiralis* larval invasion.

## Introduction

Trichinellosis is a globally distributed foodborne parasitic zoonosis and poses a significant threat to public health [1,2]. *Trichinella spiralis* infection occurs when the mammals including humans consume raw or undercooked meat infected with *T*. *spiralis* muscle larvae (ML). When the infected meat is digested in the host’s stomach, the ML are released and activated to the intestinal infective larvae (IIL) by bile and intestinal contents. Then, the IIL penetrate to intestinal epithelial cells (IECs) and develop into adult worms (AW) after four molts. After being copulated with male worms, the female worms are fertilized and subsequently give birth to newborn larvae (NBL) in the intestine [3]. Therefore, larval invasion of intestinal mucosa is a vital procedure in *T*. *spiralis* infection [4–6]. However, the absence of oral appendages and spicules in *T. spiralis* larvae suggests that gut epithelial invasion is unlikely to be caused by mechanical penetration by the larvae themselves. Instead, it is more likely to result from the degradation and destruction of gut epithelium by various proteases present in the IIL surface or their excretory-secretory proteins (ESP) [7,8]. Nevertheless, the underlying mechanism of larval invasion of gut mucosa is still not fully understood.

Some studies showed that *T*. *spiralis* larvae released their ESP to participate in the interaction with the host and immune escape to ensure their survival in hosts [9]. *T*. *spiralis* ESP have been found to take part in some vital pathological processes such as inflammatory reaction, destroying intestinal mucosal barrier and inducing epithelial apoptosis [10–12]. *T. spiralis* infection induces the apoptosis of gut mucosal epithelial cells during intestinal infection stage [13,14].

Our previous study indicated that a *T*. *spiralis* serine protease TsSPc (GenBank: U62659.1) was identified in IIL surface proteins and ESP [15,16]. Recombinant plasmid pQE-80L/TsSPc was prepared, and rTsSPc with His tag was expressed and identified in our laboratory [6]. TsSPc as a secretory serine protease was specifically bound to the RACK1 receptor and activating MAPK/ERK1/2 pathway in gut epithelium, disrupted the Caco-2 monolayer barrier function and promoted larval invasion. However, when anti-rTsSPc antibodies and TsSPc-specific dsRNA were used in the *in vitro* invasion assay, only partial larval invasion was suppressed; After the inhibitors of RACK1 receptor and ERK1/2 pathway were preadministered in mice, larval invasion of gut mucosa partly impeded, but *T. spiralis* infection was not completely interdicted [17]. The results suggested that the mechanism of TsSPc damaging gut epithelial integrity and mediating larval invasion was not fully clear. Larval invasion might be involved in other mechanisms. It is hypothesized that TsSPc may be involved in the cellular apoptosis of intestinal epithelium triggered by *T. spiralis* infection, and the detailed mechanism requires further investigation.

Furthermore, intestinal epithelial barrier plays an important role in the host defense against pathogen invasion. It is known that many pathogens consisting of virus, bacteria and parasites (such as *Giardia* and *Entamoeba*) disrupt intestinal barrier function by inducing IEC apoptosis [18,19]. Recent researches showed that exosomes and exosome-delivered miR-153 from *T. spiralis* larvae induce apoptosis and compromise the integrity of intestinal epithelial barrier [20,21]. Our previous findings indicated that *T*. *spiralis* ESP disrupted intestinal epithelial barrier and promoted larval invasion by inducing cell apoptosis [22], but the relationship between gut epithelial apoptosis and barrier integrity in *T. spiralis* infection remains unclear, and the role and mechanism of gut epithelial apoptosis in disrupting gut epithelial integrity and mediating larval invasion have not been elucidated.

Our previous results of GST pull-down assay and mass spectrometry showed that rTsSPc was capable of binding with phosphoglycerate mutase family member 5 (PGAM5) in Caco-2 cells, PGAM5 is a critical molecule involved in necrosis, apoptosis, and autophagy and participates in cell apoptosis [17,23]. The results suggested that rTsSPc might be involved in gut epithelial apoptosis and regulated the tight junctions (TJs) expression, thereby damaged gut epithelial integrity through binding PGAM5 receptor in gut epithelium. In this study, to further validate the interaction between rTsSPc and PGAM5, the IFT co-localization, GST pull-down, and co-immunoprecipitation (Co-IP) assays were used to investigate and verify the binding and interaction between rTsSPc and PGAM5, and the effect of their interaction on gut epithelial integrity.

Therefore, the main objective of this study was to investigate whether TsSPc induces gut epithelial apoptosis, disrupts epithelial integrity and promotes larval invasion during *T*. *spiralis* infection, and further identify the key protein molecules involved in the apoptosis process. Our findings will not only offer valuable insights into the invasion mechanism and pathogenesis of *T*. *spiralis*, but also provide new strategies for prevention and control of *T*. *spiralis* infection.

## Materials and methods

### Ethics statement

This study was conducted in accordance with the National Guidelines for Laboratory Animal Welfare (Ministry of Science and Technology, People’s Republic of China, 2006). All animal experimental procedures in this study were authorized by the Life Science Ethics Committee of Zhengzhou University (No. ZZUIRB GZR 2023–1379).

### Parasite and animals

The parasite used in this study was *Trichinella spiralis* (ISS534), which was isolated from naturally infected pigs in Henan Province of China and passaged in 6–8 weeks old BALB/c mice [24], and the mice were provided by the Experimental Animal Center of Zhengzhou University, Henan Province, China.

### Preparation of recombinant TsSPc protein and anti-rTsSPc serum

In our previous study, full-length TsSPc cDNA sequence was cloned into pQE-80L to construct recombinant expression plasmid pQE-80L/TsSPc [6]. The pQE-80L/TsSPc were expressed in *E. coli* BL21(DE3) (Novagen, USA) under induction of 0.5 mM isopropyl β-D-1-thiogalactopyranoside (IPTG) (Sangon Biotech, China) for 6 h at 37°C and purified by a Ni-NTA-Sefinose resin that allows purifications of the rTsSPc protein containing his tags (Sangon Biotech, China). Finally, rTsSPc was refolded by dialysis and renatured methods as previous reports in our laboratory. Briefly, the denatured rTsSPc in supernatant was refolded by sequential dialysis against buffer C (55 mM Tris-HCl, pH 8.2, 264 mM NaCl, 11 mM KCl, 0.055% PEG 3350, 8 M urea) and then against buffers with decreasing urea concentrations (8 M, 4 M, 2 M, 1 M, 0 M), each for 3 h at 4°C. After urea removal, disulfide bond formation was favored by adding 1 mM reduced and 5 mM oxidized glutathione, followed by dialysis for 3 h at 4°C. Finally, the solution was dialyzed against buffer D (50 mM Tris-HCl, pH 7.5, 100 mM NaCl, 10 mM CaCl_2_), centrifuged to remove any precipitated protein, and stored at 4°C [6,25]. Moreover, three enzyme active sites at 83 aa (His), 139 aa (Asp) and 229 aa (Ser) of TsSPc were mutated to inactive amino acid (Ala), according to the results of predicted 3-dimensional structure of TsSPc protein in our previous reports, the mutant rTsSPc (MTsSPc) was expressed and purified in our laboratory [6]. The enzymatic activity of rTsSPc and the inactivity of MTsSPc were confirmed by using the substrate BAEE in our laboratory [6].

To perform the GST pull-down assay, the rTsSPc with a GST tag expressed from pGEX-4T-1/TsSPc was also prepared. The pGEX-4T-1/TsSPc was expressed in *E. coli* Origami (Novagen, USA), induced with 0.5 mM IPTG for 3 d at 16°C and purified with GST-Sefinose Resin 4FF (Settled Resin) (Sangon Biotech, China). rTsSPc protein concentration was determined by the Bradford method and aliquoted and stored at -80°C [17].

The anti-rTsSPc serum was obtained as described before [6]. In brief, fifteen BALB/c mice were each subcutaneously immunized with 20 μg of rTsSPc emulsified in complete Freund’s adjuvant, and subsequently received two booster immunizations with 20 μg of rTsSPc emulsified in incomplete Freund’s adjuvant at 2-week intervals. Two weeks after the final immunization, tail blood was collected from the immunized mice to prepare anti-rTsSPc serum.

### Cell culture

Human colorectal adenocarcinoma cell line (Caco-2 cells) was purchased from Cell Resource Center of Shanghai Institute of Biological Sciences, Chinese Academy of Sciences [26]. Caco-2 cells in passage 30–35 were grown in 25 cm^2^ cell culture-flasks (NEST, China) and cultured with DMEM medium (Servicebio, China) containing 10% fetal bovine serum (FBS, Viva cell Bioscience, China), 100 μM non-essential amino acids (Solarbio, China), 100 µg/ml streptomycin and 100 U/ml penicillin at 37°C under 5% CO_2_ [27].

### Assay of cell viability and apoptosis

The endotoxin in the rTsSPc protein was removed using a Protein Endotoxin Removal Kit (Beyotime, China) and the endotoxin concentration were tested by Chromogenic LAL Endotoxin Assay Kit (Xiamen Bioendo Technology, China) [28]. The results indicated that the endotoxin levels of rTsSPc proteins solution were < 0.05 EU/ml, suggesting a minimal influence on the cell viability, TEER and FD-4 permeability of Caco-2 monolayers [29]. To assess the rTsSPc effect on Caco-2 cell viability, cell counting kit 8 (CCK-8) assay (Targetmol, USA) was performed as previous work [30]. In brief, 3000 cells/100 μl/well were seeded in a 96 well-plate and cultured for 24 h. Then the cells were treated with 100 μl/ well DMEM containing 20 μg/ml rTsSPc as the rTsSPc group and cultured at 37°C under 5% CO_2_; meanwhile, the DMEM medium in which rTsSPc was replaced by PBS was used as the PBS group [17]. After being incubated for 0, 3, 6, 12, 24 and 48 h, 10 μl/well CCK-8 solution was added and incubated for another 1 h. The absorbance values were measured with a plate reader (SpectraMax i3x, Molecular Devices, USA) at 450 nm. Cell viability = (OD values of rTsSPc group - OD values of blank)/ (OD values of PBS group - OD values of blank) ×100% [31].

Additionally, apoptotic cell nucleus was stained with DAPI (Solarbio, China) to observe the morphology changes after Caco-2 cells incubated with 20 μg/ml rTsSPc, MTsSPc or heat-inactivated rTsSPc (ΔrTsSPc, heated at 100°C for 10 min) for 12 h under a fluorescence microscope (Olympus, Japan) [21].

Furthermore, apoptotic cells were stained by using Terminal dUTP nick-end labeling (TUNEL). Apoptosis Assay Kit (Abbkine, China) was used according to the manufacturer’s protocol. Briefly, Caco-2 cells were incubated with 20 μg/ml rTsSPc, MTsSPc or ΔrTsSPc for 12 h, fixed with 4% paraformaldehyde and then incubated with TUNEL detection solution for 2 h at 37°C in dark. Next, the cell nuclei were stained with DAPI and observed under a fluorescence microscopy.

In addition, apoptotic cells were also observed with fluorescence microscope after Annexin V/PI staining by Annexin V-AbFluor 488/PI kit (Abbkine, China) and apoptotic cells with green staining were observed. Meanwhile, apoptosis was assessed using flow cytometry with the Annexin V-AbFluor 488/PI kit (Abbkine, China). Briefly, cells were harvested and washed twice with cold PBS. Subsequently, the cells were resuspended in 1 × binding buffer at a concentration of 1 × 10^6^ cells/ml. A total of 100 μl of the cell suspension was transferred to a new tube, and 5 μl of Annexin V-FITC and 2 μl of PI were added to the suspension. The cells were gently vortexed and incubated for 15 min at room temperature in the dark. After incubation, 400 μl of 1 × binding buffer was added to each tube, and the samples were analyzed by flow cytometry (FACScan; BD Biosciences, San Jose, CA) within 1 h. Data were analyzed using FlowJo software (Tree Star, USA). The percentage of apoptotic cells was determined by the sum of early apoptotic cells (Annexin V-FITC + /PI-) and late apoptotic cells (Annexin V-FITC + /PI+) [22].

### Quantitative real-time PCR (qPCR)

qPCR was performed to evaluate the relative mRNA expression of the related genes as previous studies [30,32]. The specific primer sequences for detecting target genes were synthesized by Sangon Biotech as shown in Table 1. After Caco-2 cells were incubated with 20 μg/ml rTsSPc, MTsSPc or ΔrTsSPc for 12 h, total RNAs of Caco-2 cells were extracted by TRIzol reagent (Sangon Biotech, China) and reverse-transcribed into cDNA according to the manufacturer’s protocol of PrimeScript RT reagent Kit (Servicebio, China). qPCR assay was carried out with Applied Biosystems 7500 Fast System (Life Technologies, USA) as previously reported. In brief, the cDNA amplification protocol consisted of an initial pre-conditioning step at 95°C for 30 sec, followed by 40 cycles of amplification (denaturation at 95°C for 30 sec, annealing at 60°C for 30 sec, and extension at 72°C for 20 sec). The cycle threshold (Ct) values for the genes were determined using the Applied Biosystems 7500 Fast System [33]. The GAPDH was used as an internal control and the results were calculated according to 2^−ΔΔCt^ method [34].

**Table 1:** Specific primer sequences of human gut epithelial TJs and apoptosis-related genes for qPCR.

Gene names	Primer	Sequence (5′-3′)	GenBank no.
Caspase 3	Forward	GAAATTGTGGAATTGATGCGTGA	NM_001354777.2
	Reverse	CTACAACGATCCCCTCTGAAAAA	
Caspase 8	Forward	TTTCTGCCTACAGGGTCATGC	NM_001080124.2
	Reverse	TGTCCAACTTTCCTTCTCCCA	
Caspase 9	Forward	GTTTGAGGACCTTCGACCAGCT	NM_001229.5
	Reverse	CAACGTACCAGGAGCCACTCTT	
PARP	Forward	CGGAGTCTTCGGATAAGCTCT	NM_001618.4
	Reverse	TTTCCATCAAACATGGGCGAC	
Cytochrome c	Forward	ATGAAGTGTTCCCAGTGCCA	NM_018947.6
	Reverse	CTCTCCCCAGATGATGCCTTTG	
Bax	Forward	TCAGGATGCGTCCACCAAGAAG	NM_001291428.2
	Reverse	TGTGTCCACGGCGGCAATCATC	
Bcl-2	Forward	ATCGCCCTGTGGATGACTGAGT	NM_000633.3
	Reverse	GCCAGGAGAAATCAAACAGAGGC	
ZO-1	Forward	CGGTCCTCTGAGCCTGTAAG	NM_001330239.4
	Reverse	GGATCTACATGCGACGACAA	
E-cadherin	Forward	GCCTCCTGAAAAGAGAGTGGAAG	NM_131820.1
	Reverse	TGGCAGTGTCTCTCCAAATCCG	
Occludin	Forward	ATGGCAAAGTGAATGACAAGCGG	XM_026274194.1
	Reverse	CTGTAACGAGGCTGCCTGAAGT	
Claudin-1	Forward	GTCTTTGACTCCTTGCTGAATCTG	NM_021101.5
	Reverse	CACCTCATCGTCTTCCAAGCAC	
PGAM5	Forward	TCGTCCATTCGTCTATGACGC	NM_001170543.2
	Reverse	GGCTTCCAATGAGACACGG	
GAPDH	Forward	TGTGTCCGTCGTGGATCTGA	NM_002046.7
	Reverse	TTGCTGTTGAAGTCGCAGGAG	

### Western blotting

To investigate the effect of rTsSPc on the expression of apoptosis proteins in intestinal epithelial cells, Western blotting assay was performed as reported previously [35,36]. After being treated with 20 μg/ml rTsSPc, MTsSPc or ΔrTsSPc for 12 h, Caco-2 cellular soluble proteins were extracted by RIPA buffer. Protein samples were resolved on 12% Tris-Glycine gels and separated by SDS-PAGE, transferred onto polyvinylidene fluoride (PVDF) membranes (Millipore, USA). The membranes were blocked with 5% skim milk dissolved in TBST (Tris-buffered saline containing 0.05% Tween 20) for 1 h at room temperature, cut into strips which were incubated overnight at 4°C with primary antibodies against: Tubulin (1:5000, Abmart, China), cl-Caspase 3 and Caspase 3 (1:500, Abmart, China), cl-Caspase 8 and Caspase 8 (1:5000, Proteintech, China), cl-Caspase 9 and Caspase 9 (1:5000, Proteintech, China), cl-PARP and PARP (1:500, Abmart, China), Bcl-2 (1:500, Abmart, China), Bax (1:1000, Abmart, China), Cytochrome C (1:1000, Abways, China), PGAM5 (1:100, Santa cruz, USA), ZO-1 (1:1000, Servicebio, China), E-cadherin (E-cad, 1:20000, Abcam, UK), Occludin (2 μg/ml, Invitrogen, USA), and Claudin-1 (2 μg/ml, Invitrogen, USA). After washing with TBST three times, the strips were incubated with secondary antibodies (goat anti-rabbit or anti-mouse IgG-HRP, 1:10000, Sangon biotech, China) for 1 h at room temperature. After washing with TBST, the strips were probed by a chemiluminescence image system (Tanon, China) using enhanced chemiluminescence (ECL) reagent (Meilunbio, China). The intensities of protein bands were quantified by ImageJ software and normalized using Tubulin as an internal control, as tubulin as a major cytoskeletal protein remains unchanged in experimental manipulations, it provides a stable baseline for accurate quantification of protein expression levels of target genes [11,37,38].

### Measurement of trans-epithelial electrical resistance (TEER) and paracellular permeability of Caco-2 monolayer

The IIL ESP disrupted intestinal epithelial barrier integrity via inducing cell apoptosis [22]. TsSPc in IIL surface proteins and ESP has been confirmed to be capable of disrupting gut epithelial barrier [17], suggesting that TsSPc might induce gut epithelial apoptosis which involved in the destruction of gut epithelial integrity and barrier functions. To confirm this hypothesis, an apoptosis inhibitor Z-VAD-FMK was used to pretreat Caco-2 cells for inhibiting apoptosis, and then the monolayer barrier integrity was assayed after rTsSPc treatment.

Caco-2 cells were grown on a trans-well insert (0.4 μm pore size, 6.5 mm diameter, BIOFIL, China). The TEER of Caco-2 monolayer was monitored by a Millicell-ERS volt-ohmmeter (Millipore, USA). TEER = (measured value - blank value) × film bottom area. When the resistance was above 300 Ω·cm^2^, Caco-2 monolayer was pretreated with 120 μM Z-VAD-FMK (APE × BIO, USA), an apoptosis inhibitor for 1 h [39,40]. Then, 20 μg/ml rTsSPc was added into the insert. After incubation for 18 h, the TEER was measured again. TEER value was normalized to its initial value before treatment and expressed as TEER (%) [41,42].

Caco-2 monolayer paracellular permeability was assessed using FITC-dextran (MW: 4 kDa, FD-4, Sigma, USA). Caco-2 cells were cultured on trans-well inserts. After pretreating with 120 μM Z-VAD-FMK for 1 h, 2 μM LFHP-1c (a PGAM5 inhibitor) for 9 h or 2 μM LFHP-1c for 9 h + 120 μM Z-VAD-FMK for 1 h at 37°C, then 20 μg/ml rTsSPc was added into the inserts and incubated for 18 h [43]. Then 0.5 mg/ml FD-4 dissolved in DMEM medium were added into the apical side of the monolayers [44]. Meanwhile, medium without FD-4 was added to the basal chamber and incubated at 37°C for 2 h [45,46]. The DMEM medium in the basal chamber was collected and the absorbance value at 485 nm-520 nm was examined by a microplate reader (Molecular Devices, USA) [26].

### Immunofluorescence test (IFT)

Caco-2 cells grown on glass coverslips to confluence were pretreated with 120 μM Z-VAD-FMK for 1 h, 2 μM LFHP-1c (a PGAM5 inhibitor) for 9 h or 2 μM LFHP-1c for 9 h + 120 μM Z-VAD-FMK for 1 h at 37°C and then incubated with 20 μg/ml rTsSPc for another 12 h. After washing with PBS, the cell monolayers were fixed in 4% paraformaldehyde at room temperature for 30 min and permeabilized with 0.25% (v/v) Triton X-100 at room temperature for 10 min. After washing with PBS, the monolayers were blocked with 1% bovine serum albumin (BSA) for 1 h at 37°C. Then coverslips were incubated with primary antibodies against the TJs diluted in 1% BSA overnight at 4°C: ZO-1 (1:500, Servicebio, China), E-cad (1:500, Abcam, UK), Occludin (1:100, Invitrogen, USA) and Claudin-1 (1:100, Invitrogen, USA). Following washes with PBS, the coverslips were incubated with CY-3-labeled anti-rabbit antibody (1:100, Servicebio, China) for 1 h at 37°C in dark, following by a DAPI for nucleus staining at room temperature for 10 min. The coverslips were mounted on glass slides and subjected to be observed and imaged under a fluorescence microscopy (Olympus, Japan) as reported previously [47]. The fluorescence intensities were quantitated by ImageJ software [26].

### IFT co-localization analysis of rTsSPc and PGAM5

Caco-2 cells grown on a glass slides were treated with 20 μg/ml rTsSPc and IIL ESP for 12 h at 37°C and fixed with 4% paraformaldehyde at room temperature for 30 min, followed by permeabilization with 0.25% (v/v) Triton X-100 at room temperature for 10 min. After washing with PBS, the Caco-2 monolayers were blocked with 1% BSA for 1 h at 37°C. Subsequently, the monolayers were incubated with primary antibodies: anti-rTsSPc serum (1:10) and anti-PGAM5 antibody (1:100, Abmart, China) overnight at 4°C. And then, the monolayers were incubated with secondary antibodies: CY3-labeled goat anti-rabbit IgG and Fluor-488-labeled goat anti-mouse IgG at 37°C in the dark for 1 h. The nuclei were counterstained with DAPI, and the slides were mounted with 30% glycerol. Finally, the cells were observed and imaged under a fluorescence microscope [5,34].

### GST pull-down assay

To confirm the binding and interaction of rTsSPc and PGAM5 in Caco-2 cells, a GST pull-down assay was carried out as reported previously [17]. In brief, GST-rTsSPc and GST tag protein (negative control) was firstly immobilized to GSH resins through co-incubating for 2 h at 4°C, the GSH resins was washed by binding buffer (140 mM NaCl, 10 mM KCl, 4.2 mM Na_2_HPO_4_ and 2 mM KH_2_PO_4_), and then resuspended in binding buffer. The Caco-2 cell lysates extracted by RIPA buffer was co-incubated with GST-rTsSPc and GST tag protein immobilized to GST resins for 2 h at 4°C, after washes by binding buffer. The protein samples were separated by SDS-PAGE with 12% Tris-Glycine gels, and transferred onto PVDF membranes. For Western blot analysis, primary antibodies used included anti-PGAM5 antibody (1:100, Santa cruz, USA), anti-rTsSPc serum (at 1:100 dilution, prepared in our laboratory) and anti-GST antibody (1:1000, Servicebio, China), and incubated with membranes at 4°C overnight. Secondary antibodies were HRP-conjugated goat anti-rabbit IgG and goat anti-mouse IgG (both at 1:10,000 dilution, Sangon biotech, China). The membranes were incubated at 37°C for 1 hour, washed with TBST for three times, and then colored with enhanced chemiluminescence (ECL) reagent (Meilunbio, China) to visualize the reactive bands [34].

### Co-IP assay

Caco-2 cells were treated with 20 μg/ml rTsSPc for 12 h at 37°C and subsequently lysed in cell lysis buffer containing 30 mM Tris-HCl (pH 7.4), 150 mM NaCl and 1% Triton X-100, supplemented with protease inhibitor cocktail (PMSF+E-64 + pepstatin+leupeptin+aprotinin+ bestatin) which was premixed by the supplier (Sangon Biotech, China). The lysate was then subjected to immunoprecipitation using Protein A/G beads (Santacruz, USA) coated with anti-rTsSPc serum at 4°C overnight. Immune complexes were detected on Western blotting with an anti-PGAM5 antibody (1:100, Santa cruz, USA) and anti-rTsSPc serum (1:100) [48].

### siRNA synthesis and transfection

The siRNAs consisting of siPGAM5 (human) and scramble siRNA (negative control for siRNA, NC-siRNA) were synthesized by Sangon biotech (Shanghai, China). The sequences were as follows: siPGAM5 (sense): 5′-CUGUGCAGUAUUACGAAGA (dT)(dT)-3′, antisense: 5′-UCUUCGUAAUACUGCACAG (dT) (dT)-3′ [49]; NC-siRNA: 5′-UUCUCCGAACGUGUCACGU (dT) (dT)-3′, antisense: ACGUGACACGUUCGGAGA A(dT) (dT). The 200 nM siRNA and NC-siRNA were transfected into Caco-2 cells grown on a 6-well cell culture plate using RNATransMate (Sangon, China) as manufacturer’s protocol, the medium was changed after incubation for 6 h, incubated for 48 h and stimulated with 20 μg/ml rTsSPc for 12 h.

### The *in vitro* larval invasion assay

To investigate the effect of inhibiting PGAM5 and apoptosis on *T. spiralis* invasion of intestinal epithelium, an *in vitro* larval invasion assay was conducted as previously described [50,51]. Muscle larvae (ML) were isolated from infected muscle tissue of mice infected with *T. spiralis* at 42 days post infection by artificial digestion using 1% pepsin (1:3000) and 1% hydrochloric acid diluted in saline, then activated to the IIL by incubation at 37°C for 2 h with 5% porcine bile obtained from a slaughter house and diluted in saline. Caco-2 monolayers were first pretreated with siPAGM5, Z-VAD-FMK (an apoptosis inhibitor) and LFHP-1c (a PGAM5 receptor inhibitor which can bind to PGAM5 and inhibit the activity of PGAM5), followed by incubating with or without rTsSPc, and then 100 IIL suspended in a 1.75% agarose mixed with RPMI-1640 medium were added and incubated for 2 h at 37°C. The number of larvae invaded into the monolayer was observed and numbered under the light microscope (Olympus, Japan). The invaded larvae exhibited the snake-like movement and migrated within the monolayer; whereas non-invaded larvae showed spirally coiled on the surface of the cell monolayer, and three replicate wells were used per condition [27,33].

### Statistical analysis

In this study, GraphPad Prism 8 was utilized for data analysis, with results depicted as means ± standard deviation (SD). For statistical comparisons, two-tailed Student’s *t*-tests or one-way ANOVA was applied, contingent upon verifying normal distribution and homogeneity of variances. In instances where these assumptions were not satisfied, we resorted to the Mann-Whitney U test and Brown-Forsythe test. Statistical significance was set at a *P* value threshold of less than 0.05.

## Results

### rTsSPc induced Caco-2 cell apoptosis

**Fig 1:**
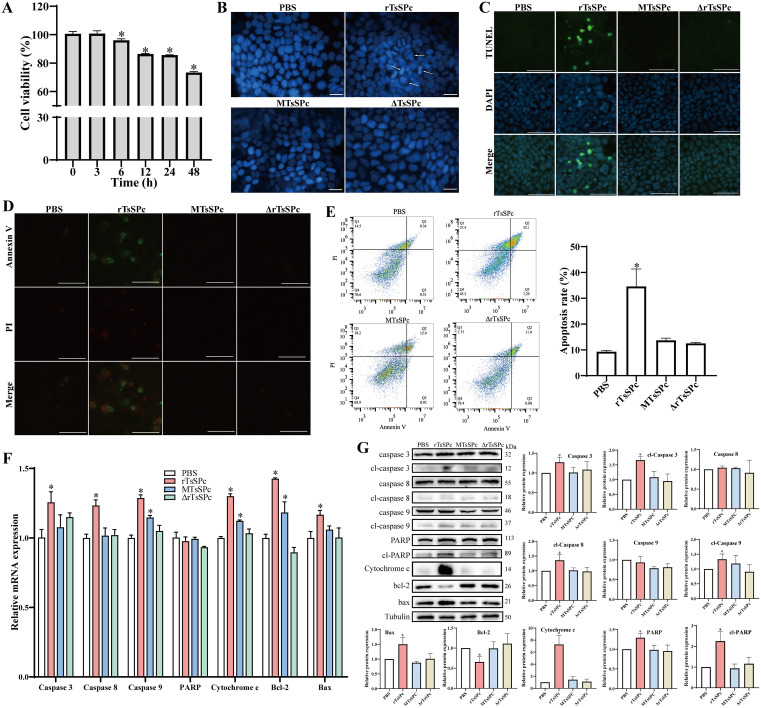
rTsSPc induced Caco-2 cell apoptosis. **A:** Viability of Caco-2 cells incubated with 20 μg/ml rTsSPc for 0, 3, 6, 12, 24 and 48 **h. B:** Caco-2 cell nuclear morphology stained by DAPI (blue), white arrows indicating the broken nuclei of apoptotic cells, scale bar: 50 μm. **C:** TUNEL staining of Caco-2 cells, the apoptotic cells were stained green, scale bar: 20 μm. **D:** Annexin V-AbFlour488 (green) and PI (red) staining of Caco-2 cells for apoptosis detection via a fluorescence microscope, scale bar: 100 μm. **E:** Flow cytometry for the apoptosis rate of Caco-2 cells. **F:** qPCR analysis of mRNA expression of apoptosis genes of Caco-2 cells. **G:** Western blotting of apoptosis protein expression of Caco-2 cells. MTsSPc: rTsSPc with enzymatic active site mutation. ΔrTsSPc: heating inactivated rTsSPc at 100°C for 10 min. Data are presented as mean ± SD (n = 3). *P < 0.05 compared to the PBS group.

The CCK-8 assay was performed to examine the effect of rTsSPc on cell viability. The results are shown in Fig 1A. When Caco-2 cells were treated with 20 μg/ml rTsSPc for 3, 6, 12, 24 and 48 h, the cell viability was 100.75% (*P* > 0.05), 96.1% (*P* < 0.001), 86.42% (*P* < 0.0001), 85.62% (*P* < 0.0001), and 73.37% (*P* < 0.0001), respectively. These results suggested that rTsSPc significantly decreased the cell activity. Therefore, 20 μg/ml rTsSPc was used to incubate with Caco-2 cells for 12 h in subsequent experiments. After treatment with rTsSPc for 12 h, the nuclei of Caco-2 cells were stained by DAPI and exhibited condensation, lobulation and fragmentation, indicating that Caco-2 cell apoptosis occurred. To investigate the relationship between rTsSPc pro-apoptotic effect and its enzymatic activity, we also observed the nuclear morphology of Caco-2 cells treated with the rTsSPc with enzymatic site mutation (MTsSPc) and heat-inactivated rTsSPc (ΔrTsSPc) (Fig 1B). The results revealed that in both MTsSPc and ΔrTsSPc groups, the cell nuclear morphology was normal, suggesting that the rTsSPc pro-apoptotic effect on Caco-2 cells is dependent on its enzymatic activity. Furthermore, the nuclei of apoptotic Caco-2 cells treated with rTsSPc were stained green fluorescence by TUNEL assay; whereas normal Caco-2 cells did not exhibit green fluorescence when Caco-2 cells were treated with MTsSPc or ΔrTsSPc (Fig 1C). These results further confirmed that rTsSPc-induced Caco-2 apoptosis and the rTsSPc pro-apoptotic effect was dependent of its enzymatic activity. To further investigate rTsSPc-induced apoptosis, rTsSPc-incubated Caco-2 cells were also stained by Annexin V and PI kit, and observed (Fig 1D). Flow cytometry was subsequently served to assess the apoptosis rate of the cells. The apoptosis rate in the rTsSPc group was significantly increased by 25.28% compared with the PBS group (*t* = 9.05, *P* < 0.0001). But in the MTsSPc and ΔrTsSPc groups, the cell apoptosis rate had no significant changes compared with the PBS group (*t*_MTsSPc_ = 1.58, *P* > 0.05; *t*_ΔrTsSPc_ = 1.15, *P* > 0.05) (Fig 1E). These results further confirmed that rTsSPc induced Caco-2 cell apoptosis and the rTsSPc pro-apoptotic effect was dependent on the rTsSPc enzymatic activity.

To further observe the influence of rTsSPc on mRNA and proteins expression levels of apoptosis genes in Caco-2 cells, qPCR and Western blotting were performed. qPCR results showed that rTsSPc significantly increased the mRNA level of Caspase 3, Caspase 8, Caspase 9, Cytochrome c, and the pro-apoptotic gene Bax in Caco-2 cells by increasing to 0.25, 0.23, 0.29, 0.30 and 0.17 fold of the PBS group, respectively, (*t*_Caspase 3_ = 4.64, *t*_Caspase 8_ = 6.85, *t*_Caspase 9_ = 13.35, *t*_Cytochrome c _= 19.98, *t*_Bax_ = 4.463, *P* < 0.01). The transcription level of anti-apoptotic gene Bcl-2 was also increased to 0.43 fold of the PBS group (*t*_Bcl-2_ = 11.83, *P* < 0.0001). However, the transcription level of PARP showed no significant change compared to the PBS group (*P* > 0.05). The transcription levels of Caspase 9, Cytochrome c and Bcl-2 in the MTsSPc group were obviously increased compared to the PBS group (*P* < 0.05), but transcription levels of Caspase 3, Caspase 8 and Bax have no significant change. In contrast, the mRNA level of these genes in ΔrTsSPc group had no significant change compared to the PBS group (Fig 1F). These results indicated that rTsSPc up-regulated the transcription levels of apoptosis-related genes in Caco-2 cells.

Western blot results showed that compared to the PBS group, treatment of Caco-2 cells with rTsSPc significantly increased the protein expression levels of apoptosis-related molecules. Specifically, the expression levels of Caspase 3 and its cleaved form cl-Caspase 3, the cleaved Caspase 8 (cl-Caspase 8), the cleaved Caspase 9 (cl-Caspase 9), the pro-apoptotic molecules Bax and Cytochrome c, as well as PARP and its cleaved form cl-PARP, were increased respectively to 0.27, 0.66, 0.35, 0.33, 0.50, 6.28, 0.29 and 1.26 fold of the PBS group (*t*_Caspase 3_ = 3.58, *P* < 0.05; *t*_cl-Caspase 3_ = 9.43, *P* < 0.001; *t*_cl-Caspase 8_ = 3.18, *P* < 0.05; *t*_cl-Caspase 9_ = 3.25, *P* < 0.05, *t*_Bax_ = 7.32, *P* < 0.05; *t*_Cytochrome c_ = 7.46, *P* < 0.01; *t*_PARP_ = 4.63, *P* < 0.01; *t*_cl-PARP_ = 4.73, *P* < 0.01). Conversely, the expression level of anti-apoptotic pro*t*ein Bcl-2 was decreased to 0.66 fold of *t*he PBS group (*t*_Bcl-2_ = 4.45, *P* < 0.05). Notably, rTsSPc had no significant effect on the expression levels of Caspase 8 and Caspase 9 (*P* > 0.05). The effects of MTsSPc and ΔrTsSPc on expression levels of apoptosis-related pro*t*eins were not statistically significant compared to the PBS group (*P* > 0.05) (Fig 1G). Therefore, the results of both mRNA and protein expression levels collectively indicated that rTsSPc upregulated and activated the expression of pro-apoptotic molecules, while downregulating and inhibiting the expression of anti-apoptotic molecules, thereby activated the apoptosis pathway and induced Caco-2 cell apoptosis.

### Apoptosis inhibitor Z-VAD-FMK protected Caco-2 monolayer integrity from rTsSPc disruption

**Fig 2:**
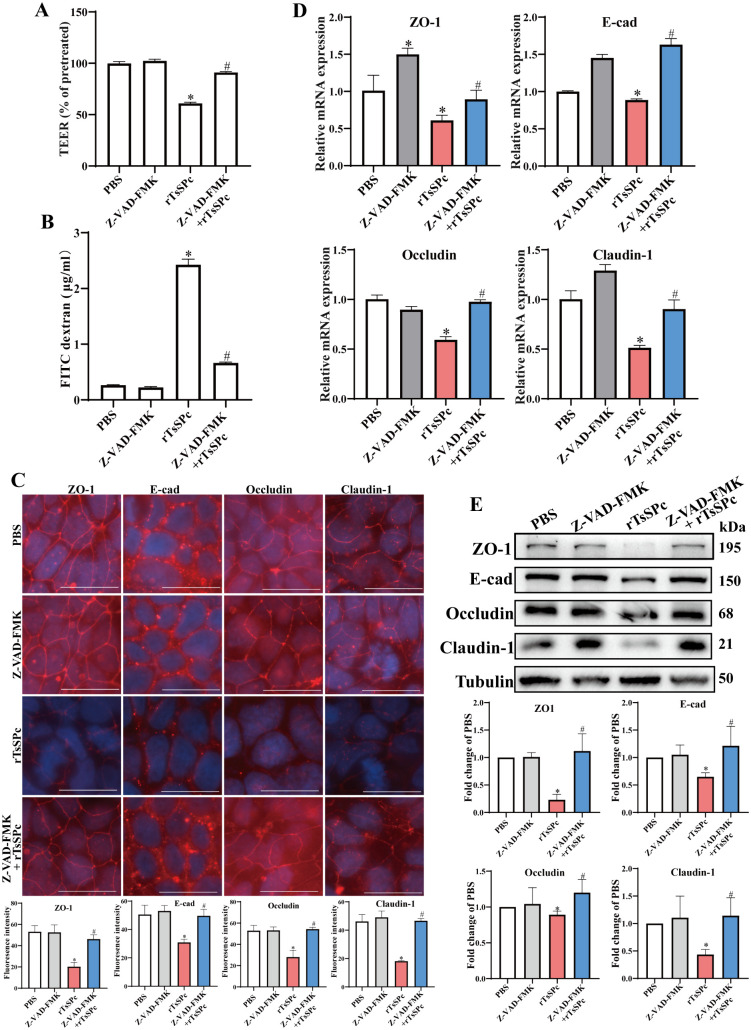
Apoptosis inhibitor abolished and restored rTsSPc-disrupted Caco-2 monolayer barrier integrity. Caco-2 cells were pretreated with 120 μM Z-VAD-FMK for 1 h and then incubated with 20 μg/ml rTsSPc for another 12 **h. A:** Z-VAD-FMK pretreatment increased the rTsSPc-reduced TEER of Caco-2 monolayer. **B:** Z-VAD-FMK pretreatment decreased the rTsSPc-increased FD-4 flux. **C:** IFT analysis of TJs proteins expression of Caco-2 monolayer. Up: images, Down: quantitative analysis of fluorescence intensities. Scale bar: 20 μm. **D:** qPCR analysis of TJs mRNA expression of Caco-2 cells normalized to DAPDH. **E:** Western blotting analysis of TJs protein expression of Caco-2 cells. Data are presented as mean ± SD (n = 3). *P < 0.05 compared to the PBS group, ^#^*P* < 0.05 compared to the only rTsSPc group.

The results of TEER measurement showed that compared with the PBS group, treatment of Caco-2 cells with rTsSPc led to a 39.00% TEER decrease (*t* = 30.55, *P* < 0.0001). However, the TEER in the Z-VAD-FMK + rTsSPc group was increased by 49.43% compared with the rTsSPc group (*t* = 34.08, *P* < 0.0001) (Fig 2A). Meanwhile, the amount of FITC-dextran permeated through the cell monolayer to the lower chamber in the rTsSPc group was 8.22 times higher than that in the PBS group (*t* = 37.72, *P* < 0.0001). Moreover, the permeation of FITC-dextran in the Z-VAD-FMK + rTsSPc group was decreased to 0.27 folds of the rTsSPc group (*t* = 30.59, *P* < 0.001) (Fig 2B). These results suggested that rTsSPc increased the paracellular permeability of Caco-2 monolayer; whereas the apoptosis inhibitor Z-VAD-FMK partially abolished the rTsSPc effect and decreased the paracellular permeability.

The IFT results revealed that after co-incubation of Caco-2 monolayers with rTsSPc, the TJs (ZO-1, E-cad, Occludin and Claudin-1) exhibited fragmentation and even disappearance among Caco-2 cells (Fig 2C). The expression levels of four TJs were significantly reduced compared to the PBS group (*P* < 0.01). However, after pretreatment with the apoptosis inhibitor Z-VAD-FMK, the expression levels of ZO-1, E-cad, Occludin and Claudin-1 in the Z-VAD-FMK + rTsSPc group were remarkably increased compared to the rTsSPc group (*P* < 0.01).

The qPCR results demonstrated that compared to the PBS group, rTsSPc treatment significantly down-regulated the mRNA expression levels of ZO-1, E-cad, Occludin and Claudin-1 of Caco-2 monolayer (*P* < 0.05) (Fig 2D). Notably, pre-treatment with the apoptosis inhibitor Z-VAD-FMK significantly increased the mRNA expression levels of these TJs proteins in the Z-VAD-FMK + rTsSPc group compared to the rTsSPc group. The mRNA expression levels of ZO-1, E-cad, Occludin and Claudin-1 were obviously increased (*P* < 0.05). These findings suggested that rTsSPc down-regulated significantly the transcriptional levels of TJ genes in Caco-2 cells; whereas apoptosis inhibition by Z-VAD-FMK abolished and restored the rTsSPc-down-regulated mRNA expression levels of ZO-1, E-cad, Occludin and Claudin-1.

Western blot results revealed that the protein expression levels of ZO-1, E-cad, Occludin and Claudin-1 in rTsSPc-treated Caco-2 monolayers were significantly reduced compared to the PBS group (*P* < 0.05) (Fig 2E). However, pre-treatment with apoptosis inhibitor Z-VAD-FMK significantly increased the expression levels of ZO-1, E-cad, Occludin and Claudin-1 in the Z-VAD-FMK + rTsSPc group compared to the rTsSPc group (*P* < 0.05).

These above results indicated that rTsSPc increased paracellular permeability, down-regulated the expression levels of TJs (ZO-1, E-cad, Occludin and Claudin-1), as a result, compromised the barrier function of intestinal epithelial monolayer. However, the apoptosis inhibitor Z-VAD-FMK counteracted the rTsSPc-increased paracellular permeability and rTsSPc-downregulated the TJs expression. In other words, apoptosis inhibitor suppressed Caco-2 apoptosis and restored rTsSPc- disrupted intestinal epithelial integrity.

### rTsSPc interacted with PGAM5 in Caco-2 cells and increased its protein expression

**Fig 3:**
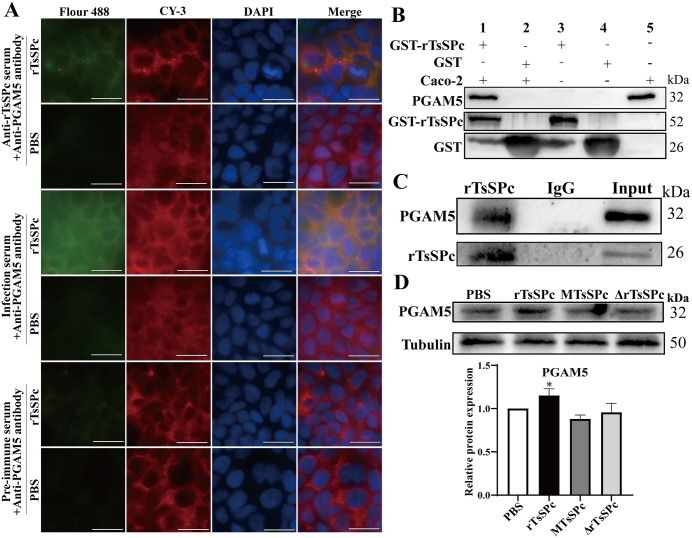
rTsSPc interacted with PGAM5 and increased its protein expression in Caco-2 cells. **A:** IFT analysis of co-localization of rTsSPc and PGAM5. After treatment with rTsSPc for 12 h, Caco-2 cells were probed overnight with primary antibodies (anti-PGAM5 antibody, anti-rTsSPc serum, infection serum or pre-immune serum). The cells were subsequently incubated with secondary antibodies (goat anti-mouse IgG conjugated to Flour 488 and goat anti-rabbit IgG conjugated to CY-3). Cell nuclei were stained with DAPI. PGAM5 was labeled red, rTsSPc was exhibited green, and nuclei were stained blue. Scale bar: 20 μm. **B:** GST pull-down and Western blotting of binding between rTsSPc and PGAM5. Lane 1: GST-rTsSPc + Caco-2 cell lysate; lane 2: GST tag protein + Caco-2 cell lysate; lane 3: Input of GST-rTsSPc protein; lane 4: Input of GST tag protein; lane 5: Input of PGAM5 from Caco-2 cell lysate. **C:** Co-IP and Western blotting of interaction between rTsSPc and PGAM5 in Caco-2 in natural condition. After co-incubation of Caco-2 cells with rTsSPc, cell lysates were collected and incubated overnight with Protein A/G beads and anti-rTsSPc immune serum or normal mouse IgG. Subsequently, Western blotting was performed using antibodies against PGAM5. rTsSPc: Co-IP complex (rTsSPc, anti-rTsSPc immune serum and cell lysates from Caco-2 incubated with rTsSPc); IgG: normal mouse IgG; Input: the cell lysates from Caco-2 incubated with rTsSPc. **D:** Western blotting of expression of PGAM5 in Caco-2 cells. Data are presented as mean ± SD (n = 3). *P < 0.05 compared to the PBS group.

The IFT results of rTsSPc and PGAM5 co-localization in Caco-2 cells are shown in Fig 3A. After treatment with rTsSPc for 12 h, Caco-2 cells were subjected to immunofluorescence staining with anti-rTsSPc serum and anti-PGAM5 antibody. The results revealed that rTsSPc exhibited green fluorescence, PGAM5 exhibited red fluorescence, and the cell nuclei showed blue fluorescence. After image overlay analysis, the co-localization of rTsSPc and PGAM5 was observed in the cytoplasm, appearing as yellow fluorescence. This finding suggested the specific binding and interaction between rTsSPc and PGAM5.

To further confirm the direct binding between rTsSPc and PGAM5 in Caco-2 cells, GST pull-down and Western blotting assay were carried out. As shown in Fig 3B, when rTsSPc was conjugated to GST beads and subsequently incubated with soluble Caco-2 cell proteins, PGAM5 was successfully pulled down. In contrast, the GST tag alone failed to pull down PGAM5 from the cell lysates. These results indicated that rTsSPc interacted directly with PGAM5. Furthermore, Co-IP assay was also performed using anti-rTsSPc immune serum and normal mouse IgG to capture PGAM5 in rTsSPc-treated Caco-2 cell lysate. The results revealed that PGAM5 in Caco-2 cells was captured by anti-rTsSPc serum (Fig 3C). In contrast, normal mouse IgG failed to capture PGAM5. In addition, Western blotting showed that rTsSPc significantly increased the PGAM5 expression in Caco-2 cells by 0.15 fold, compared with PBS group (Fig 3D). These findings indicated that rTsSPc was bound directly with PGAM5 and up-regulated its expression in Caco-2 cells.

### Knockdown of PGAM5 alleviated the rTsSPc-disrupted intestinal epithelial barrier

**Fig 4:**
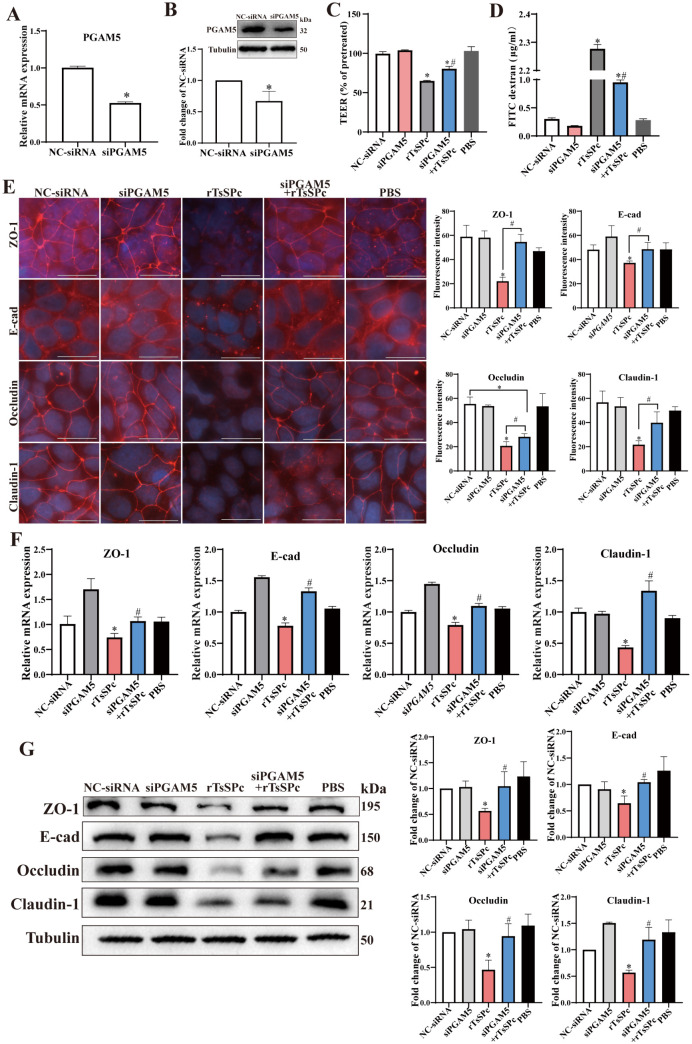
PGAM5 knockdown prevented rTsSPc from disrupting Caco-2 monolayer integrity. The knockdown efficiency of PGAM5 in Caco-2 cells by siPGAM5 transfection was confirmed by qPCR (**A**) and Western blotting analysis **(B)**. PGAM5 knockdown significantly inhibited rTsSPc-induced reduction in TEER (**C**) and increase in FD-4 flux **(D)**. **E:** IFT of expression of TJs (ZO-1, E-cad, Occludin and Claudin-1) in Caco-2 cells treated by siPGAM5 and/or rTsSPc, scale bar: 20 μm. **F:** qPCR analysis of transcription level of TJs (ZO-1, E-cad, Occludin and Claudin-1) normalized to GAPDH, relative to NC-siRNA group. **G:** Western blotting of expression of TJs (ZO-1, E-cad, Occludin and Claudin-1) in Caco-2 cells treated by siPGAM5 and/or rTsSPc. Data were presented as mean ± SD (n = 3). *P < 0.05 compared to the NC-siRNA group, ^#^*P* < 0.05 compared to the rTsSPc group.

To evaluate the effect of PGAM5 on the rTsSPc disrupting Caco-2 monolayer barrier function, siRNA transfection technology was used to knockdown PGAM5 in Caco-2 cells which were then incubated with rTsSPc, followed by measuring Caco-2 monolayer barrier integrity. The knockdown efficiency of PGAM5 in Caco-2 cells by siRNA transfection was confirmed by qPCR and Western blotting, the results revealed that siPGAM5 effectively decreased the mRNA and protein expression to respectively 0.52 fold (*t* = 31.98, *P* < 0.001) and 0.67 fold (*t* = 3.66, *P* < 0.05) of NC-siRNA group (Fig 4A and 4B).

The barrier function of PGAM5-knockdown Caco-2 monolayer cultured with siPGAM5 and further incubated with rTsSPc was ascertained by using TEER and FD-4 flux. As shown in Fig 4C, compared with the NC-siRNA group, rTsSPc treatment led to a 34.76% decrease of TEER (*t* = 24.26, *P* < 0.0001). However, when cells were transfected with siPGAM5 and subsequently treated with rTsSPc (e.g., the siPGAM5 + rTsSPc group), the TEER was increased by 23.70% compared to the only rTsSPc group (*t* = 8.424, *P* < 0.01). Furthermore, the amount of FITC-dextran permeated through the cell monolayer to the lower chamber in rTsSPc group was 7.47 fold higher than that of the NC-siRNA group (*t* = 141.224, *P* < 0.0001). In contrast, the permeation of FITC-dextran in the siPGAM5 + rTsSPc group was decreased to 0.42 fold of rTsSPc group (*t* = 52.57, *P* < 0.0001) (Fig 4D). These results indicated that rTsSPc increased paracellular permeability, while knockdown of PGAM5 gene in Caco-2 cells partially counteracted and reduced the rTsSPc-increased paracellular permeability.

To evaluate the role of PGAM5 in rTsSPc down-regulating the expression of TJs (ZO-1, E-cad, Occludin and Claudin-1), IFT, qPCR and Western blotting were performed. The IFT results showed that the expressions of ZO-1, E-cad, Occludin and Claudin-1 in rTsSPc group were obviously reduced compared to the NC-siRNA group (*P* < 0.05). However, in siPGAM5 + rTsSPc group, the expression levels of ZO-1, E-cad, Occludin and Claudin-1 were significantly increased, compared to the individual rTsSPc group (*P* < 0.05) (Fig 4E). These findings demonstrated that rTsSPc significantly decreased the expression of TJs (ZO-1, E-cad, Occludin and Claudin-1) in intestinal epithelia; whereas knockdown of PGAM5 partially abolished and restored the rTsSPc-reduced TJs expression.

Additionally, the qPCR results were presented in Fig 4F. Following treatment with rTsSPc, the mRNA expression levels of ZO-1, E-cad, Occludin and Claudin-1 in Caco-2 cells were evidently downregulated, compared to NC-siRNA group (*P* < 0.05). Conversely, in siPGAM5 + rTsSPc group, transcriptional levels of ZO-1, E-cad, Occludin and Claudin-1 were significantly increased, compared to only rTsSPc group (*P* < 0.05). These findings demonstrated that rTsSPc downregulated the mRNA levels of ZO-1, E-cad, Occludin and Claudin-1 in gut epithelia, while knockdown of PGAM5 gene effectively prevented rTsSPc from downregulating the transcription level of the TJs genes. Western blotting revealed that rTsSPc significantly reduced the expression of the TJs (ZO-1, E-cad, Occludin and Claudin-1), compared to the NC-siRNA group (*P* < 0.05). In contrast, in siPGAM5 + rTsSPc group, the expression levels of ZO-1, E-cad, Occludin and Claudin-1 were significantly restored and increased, compared to rTsSPc alone group (*P* < 0.05) (Fig 4G). These results suggested that rTsSPc obviously down-regulated the mRNA and protein expressions of the TJs in gut mucosal epithelium; whereas PGAM5 knockdown counteracted and restored rTsSPc-reduced the TJs expression.

Overall, these findings confirmed that rTsSPc disrupted intestinal epithelial integrity and barrier function by interacting with PGAM5, and further testified the crucial role of PGAM5 in rTsSPc disrupting intestinal epithelial integrity.

### Inhibitors of PGAM5 and apoptosis relieved rTsSPc-disrupted gut epithelial integrity

**Fig 5:**
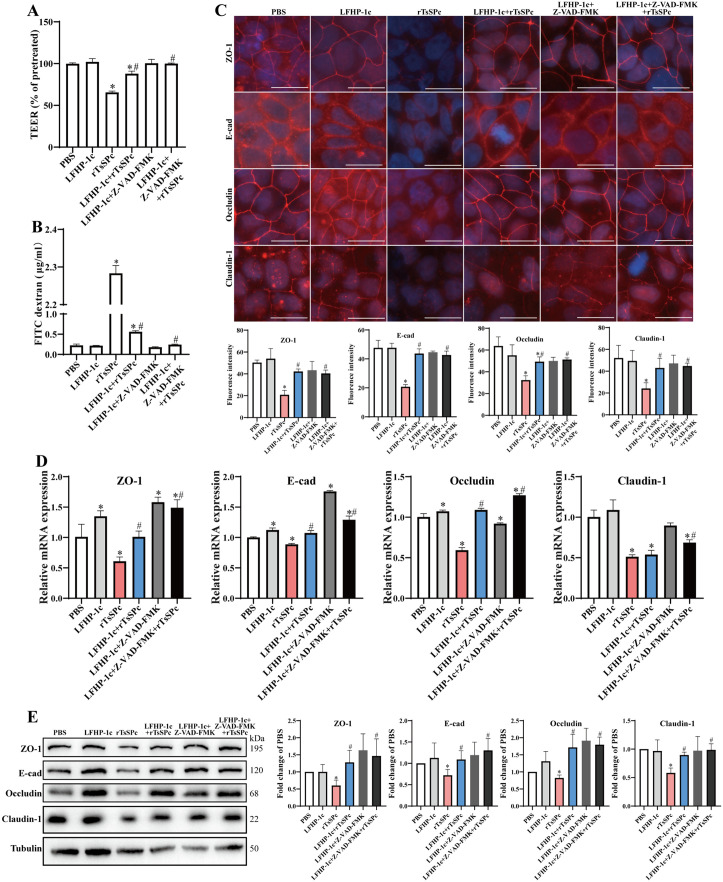
Inhibition of PGAM5 and apoptosis alleviated the rTsSPc-damaged Caco-2 monolayer barrier integrity. PGAM5 inhibitor LFHP-1c and an apoptosis inhibitor Z-VAD-FMK were used to impede rTsSPc-induced disruption of Caco-2 monolayer barrier function. To inhibit the PGAM5 and cell apoptosis, Caco-2 monolayers were pretreated with 2 μM LFHP-1c for 9 h or 2 μM LFHP-1c for 9 h + 120 μM Z-VAD-FMK for 1 h at 37°C, then incubated with rTsSPc for 12 h at 37°C. **A:** The TEER measurement of Caco-2 monolayer treated with inhibitors and rTsSPc. **B:** The amount of FITC-dextran permeated through Caco-2 monolayer incubated with inhibitors and rTsSPc. **C:** IFT of TJs expression and distribution in Caco-2 monolayer incubated with inhibitors and rTsSPc, scale bar: 20 μm. **D:** qPCR assay of the TJs transcription in Caco-2 monolayers treated with inhibitors and rTsSPc. **E:** Western blotting of TJs expression in Caco-2 monolayers after incubation with inhibitors and rTsSPc. Data were presented as mean ± SD (n = 3). *P < 0.05 compared to the PBS group, ^#^*P* < 0.05 compared to the rTsSPc alone group.

After Caco-2 cells were pretreated by PGAM5 receptor inhibitor LFHP-1c or LFHP-1c + an apoptosis inhibitor Z-VAD-FMK, then incubated with rTsSPc, The TEER measurement revealed that rTsSPc significantly decreased the TEER of Caco-2 monolayer compared to the PBS group (*t* = 31.24, *P* < 0.0001) (Fig 5A). However, pretreatment with the PGAM5 inhibitor LFHP-1c and LFHP-1c + an apoptosis inhibitor Z-VAD-FMK, then incubation with rTsSPc distinctly increased the TEER by 34.11% (*t* = 13.61, *P* < 0.0001) and 52.67% (*t* = 21.01, *P* < 0.0001), respectively, compared *t*o the rTsSPc alone group. The amount of FD-4 permeated through Caco-2 monolayer to the lower chamber in the rTsSPc group was significantly higher than the PBS group (*t* = 100.1, *P* < 0.0001) (Fig 5B). After pretrea*t*ing with LFHP-1c or LFHP-1c + Z-VAD-FMK and rTsSPc, the permeation of FD-4 was significantly decreased compared to the rTsSPc group, as decreased by 75% (*t* = 95.46, *P* < 0.0001) and 89% (*t* = 113.0, *P* < 0.0001), respectively. These results indica*t*ed tha*t* both inhibitors of PGAM5 receptor and apoptosis partially abolished and reduced the rTsSPc-increased paracellular permeability.

To investigate the effect of PGAM5 and apoptosis inhibition on the expression of rTsSPc-reduced TJs, the IFT was performed after Caco-2 cells were pretreated with PGAM5 inhibitor LFHP-1c or LFHP-1c+apoptosis inhibitor Z-VAD-FMK, and then incubated with rTsSPc. The IFT results showed that rTsSPc significantly reduced the expression of TJs (ZO-1, E-cad, Occludin and Claudin-1) in Caco-2 cells compared to the PBS group (*P* < 0.05). However, pretreatment with LFHP-1c and LFHP-1c + Z-VAD-FMK significantly restored the expression levels of ZO-1, E-cad, Occludin and Claudin-1 compared to the rTsSPc alone group (*F*_ZO-1_ = 41.40, *P* < 0.001; *F*_E-cad_ = 74.66, *P* < 0.0001; *F*_Occludin_ = 25.94, *P* < 0.01; *F*_Claudin-1_ = 10.50, *P* < 0.05). However, there was no significantly statistical difference in the TJs expression levels between the LFHP-1c group and the LFHP-1c + Z-VAD-FMK group (*P* > 0.05) (Fig 5C). These findings indicated that rTsSPc decreased the expression levels of TJs (ZO-1, E-cad, Occludin and Claudin-1), inhibition of PGAM5 receptor or apoptosis pathway impeded and restored the rTsSPc-reduced the expression levels of the TJs proteins

qPCR analysis revealed that after rTsSPc treatment, the mRNA expression of TJs in Caco-2 cells was evidently changed. Compared to PBS group, rTsSPc markedly reduced mRNA levels of the TJs ZO-1 (*t* = 3.16, *P* < 0.05), E-cad (*t* = 10.75, *P* < 0.001), Occludin (*t* = 13.50, *P* < 0.001) and Claudin-1 (*t *= 9.63, *P* < 0.001). Treatment of Caco-2 monolayer wi*t*h PGAM5 inhibitor LFHP-1c or LFHP-1c + an apoptosis inhibitor Z-VAD-FMK substantially mitigated the rTsSPc-reduced mRNA levels of TJs (ZO-1, E-cad, Occludin and Claudin-1) (*P* < 0.05). However, the Claudin-1 mRNA levels in the LFHP-1c + rTsSPc group had no significant difference compared to the rTsSPc group (*t* = 0.85, *P* > 0.05) (Fig 5D). These findings indicated that PGAM5 inhibition, particularly when combined with apoptosis pathway blockade, partially eliminated the rTsSPc-decreased mRNA expression levels of ZO-1, E-cad and Occludin, but LFHP-1c alone could not restore the rTsSPc-reduced the Claudin-1 mRNA level; however, when LFHP-1c was used in combination with the apoptosis inhibitor Z-VAD-FMK, the Claudin-1 mRNA expression level was partially restored again.

Western blotting indicated that, compared with the PBS group, rTsSPc significantly reduced the protein expression levels of ZO-1, E-cad, Occludin and Claudin-1 (*P* < 0.05) (Fig 5E). However, pre-treatment of Caco-2 cells with the PGAM5 inhibitor LFHP-1c and LFHP-1c + Z-VAD-FMK following by incubation with rTsSPc obviously increased the ZO-1 expression compared to the rTsSPc group (*t*_LFHP-1c+ rTsSPc_ = 3.09, *t*_Z-VAD-FMK +LFHP-1c + rTsSPc_ = 2.86, *P* < 0.05). Similarly, in *t*he LFHP-1c + rTsSPc group and LFHP-1c + Z-VAD-FMK + rTsSPc group, the E-cad expression levels were significantly up-regulated compared to the only rTsSPc group (*t*_LFHP-1c+rTsSPc_ = 3.48, *t*_Z-VAD-FMK+LFHP-1c+ rTsSPc_ = 3.43, *P* < 0.05). The Occludin levels in the LFHP-1c + rTsSPc group and LFHP-1c + Z-VAD-FMK + rTsSPc group was also significantly increased relative to the individual rTsSPc group (*t*_LFHP-1c + rTsSPc_ = 3.91, *P* < 0.05; *t*_Z-VAD-FMK + LFHP-1c + rTsSPc_ = 7.16, *P* < 0.01). Additionally, *t*he Claudin-1 expression level of the LFHP-1c + rTsSPc and LFHP-1c + Z-VAD-FMK + rTsSPc groups was remarkably up-regula*t*ed compared to the only rTsSPc group (*t*_LFHP-1c + rTsSPc_ = 5.55, *t*_Z-VAD-FMK + LFHP-1c + rTsSPc_ = 5.14, *P* < 0.01). However, the expression levels of ZO-1, E-cad, Occludin and Claudin-1 had no significant differences between the LFHP-1c + Z-VAD-FMK + rTsSPc group and the LFHP-1c + rTsSPc group (*P* > 0.05). These results suggested that the PGAM5 inhibitor LFHP-1c partially abolished and restored the rTsSPc-decreased expression levels of ZO-1, E-cad, Occludin and Claudin-1 in Caco-2 monolayers.

The above findings demonstrated that inhibition of the PGAM5 receptor and blocking apoptosis improved and alleviated the rTsSPc-disrupted Caco-2 monolayer integrity, and further confirmed that gut epithelial receptor PGAM5 and apoptosis were involved in the damage of gut epithelial barrier integrity resulted from rTsSPc.

### Inhibition of PGAM5 and cell apoptosis impeded larval invasion

**Fig 6:**
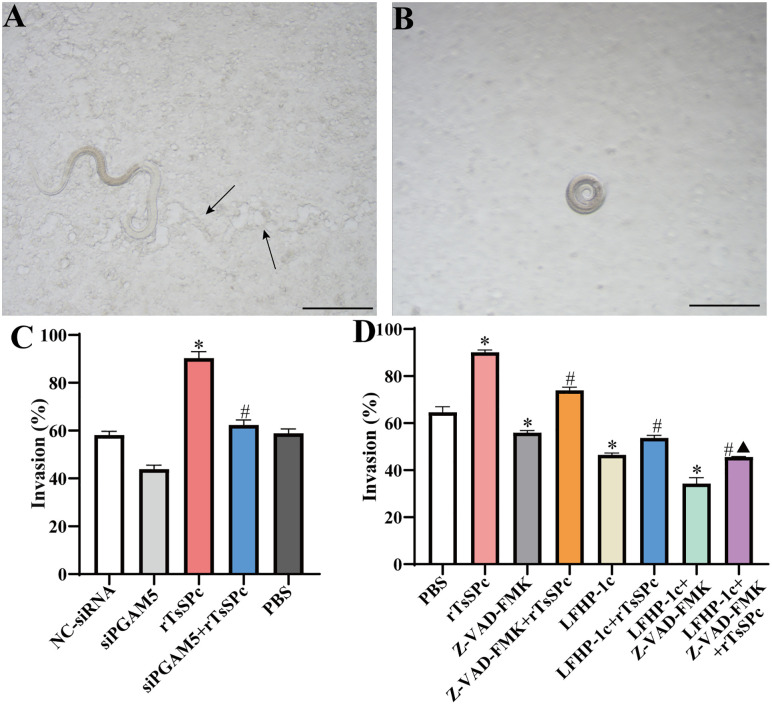
Inhibition of PGAM5 and apoptosis prevented *T. spiralis* larval invasion of Caco-2 monolayer. **A:** A larva invaded the Caco-2 monolayer with migratory traces (black arrows), scale bar: 100 μm. **B:** A non-invasion larva coiled in a spiral shape and suspended in the medium. **C:** PGAM5 knockdown of Caco-2 cells abolished and reduced rTsSPc-increased larval invasion. **D:** Pretreatment of Caco-2 monolayer with apoptosis inhibitor Z-VAD-FMK and PGAM5 inhibitor LFHP-1c obviously reduced the rTsSPc-increased larval invasion. Data were presented as mean ± SD (n = 3). *P < 0.05 compared to the NC-siRNA or PBS, ^#^*P* < 0.05 compared to the rTsSPc group, ^▲^*P* < 0.05 compared to the LFHP-1c + rTsSPc or Z-VAD-FMK + rTsSPc group.

The results of the in *vitro* larval invasion assay revealed that *T. spiralis* IIL penetrated the Caco-2 monolayer, migrated and left the migratory traces (black arrows) (Fig 6A). The non-invaded larvae were suspended in the medium and coiled in a spiral shape on the monolayer surface (Fig 6B). After Caco-2 monolayers were treated with rTsSPc, the larval invasion was increased by 55.4% compared to the NC-siRNA group *(t* = 17.56, *P* < 0.0001). However, when Caco-2 cells were transfected with siPGAM5 and then treated with rTsSPc, larval invasion was decreased by 30.9% compared to the rTsSPc alone group (*t* = 17.07, *P* < 0.0001), indica*t*ing that knockdown of PGAM5 counteracted and decreased rTsSPc-promoted larval invasion (Fig 6C). Furthermore, after Caco-2 monolayers were incubated with rTsSPc, the larval invasion was significantly increased compared to the PBS group (*t* = 5.15, *P* < 0.01). However, when Caco-2 cells were pretrea*t*ed with apoptosis inhibitor Z-VAD-FMK, the PGAM5 inhibitor LFHP-1c, or LFHP-1c + Z-VAD-FMK, then incubated with rTsSPc, larval invasion was evidently reduced, as demonstrated by a reduction of 17.94% (*t* = 19.57, *P* < 0.0001), 40.40% (*t* = 44.05, *P* < 0.0001), and 49.32% (*t* = 53.78, *P* < 0.0001), respec*t*ively, compared to *t*he rTsSPc alone group. In addition, larval invasion (45.65%) of *t*he LFHP-1c + Z-VAD-FMK + rTsSPc group was lower than 73.92% of the Z-VAD-FMK + rTsSPc group (*t* = 34.03, *P* < 0.0001) and 53.69% of LFHP-1c + rTsSPc group (*t* = 9.68, *P* < 0.001) (Fig 6D). These results suggested that inhibition of PGAM5 and apoptosis prevented the rTsSPc promotion on larval invasion of Caco-2 monolayer.

In addition, the raw data of Figs 1-6 in this study were shown in S1 Table, the fold changes and statistical results of TJs expression levels in the various groups were show in S2–S4 Tables.

## Discussion

The hosts have native defensive ability to defend the invasion of exogenous pathogens, including viruses, bacteria and parasites. However, these pathogens can dwell in the mammal hosts by inducing host cell apoptosis, thereby achieving a delicate balance between damaging host cells and their own survival [52–54]. Additionally, the IECs serve as the primary physical barrier of the gut and protect against luminal antigens, toxins, and harmful substances [55]. The TJs, as vital intercellular structures, play an essential role in maintaining the functional integrity and stability of intestinal epithelial barrier [11,56]. The TJ complex is primarily composed of zona occludens proteins (ZOs), Occludin and Claudins [57,58]. In addition, E-cad as an important composition of adjacent junctions (AJs) is also involved in maintaining epithelial barrier function [59]. Therefore, in the present study, the expression of several TJs proteins (ZO-1, E-cad, Occludin and Claudin-1) was assessed after Caco-2 monolayers were treated by rTsSPc to investigate its effect on the host intestinal epithelial barrier.

In this study, we investigated the impact of TsSPc on gut epithelial apoptosis and barrier integrity using *in vitro* Caco-2 cell model. Our results demonstrated that rTsSPc significantly reduced Caco-2 cell viability and induced apoptosis by using CCK-8, DAPI, TUNEL assays, and Annexin V/PI staining with flow cytometry. Notably, enzymatically inactive mutant (MTsSPc) and heat-inactivated rTsSPc failed to induce apoptosis, underscoring the critical role of TsSPc’s enzymatic activity in mediating apoptosis. These findings suggested that TsSPc’s pro-apoptotic function is dependent on its enzymatic activity, which disrupted gut barrier function and contributed to the invasion and pathogenesis of *T. spiralis*. Previous researches indicated that active serine proteases cleave and activate the PAR2 receptor in cells, triggering the MAPK/ERK pathway and reducing the expression level of TJs proteins (ZO-1, Occludin, Claudin-1) to impair the epithelial barrier [60,61]. In this study, the rTsSPc’s pro-apoptotic role is likely because rTsSPc cleaved and activated the downstream signaling molecules, leading to the activation of caspases that ultimately resulted in apoptosis. Our results verified that enzymatically inactive mutants (MTsSPc) and heat-inactivated rTsSPc failed to induce apoptosis, further confirming the role of rTsSPc’s enzymatic activity in inducing apoptosis.

The apparent contradiction was found between the significant upregulation of Caspase 9, Cytochrome c, and Bcl-2 mRNA and the lack of pro-apoptotic effects observed in the phenotypic assays induced by MTsSPc. Though engineered as an inactive enzyme mutant, MTsSPc may retain slight residual activity. This minimal activity could suffice to boost the transcription of apoptosis-linked genes but falls short of initiating the complete apoptotic process seen in phenotypic tests. The upregulation of these genes may reflect activation of alternative pathways by MTsSPc that, while not directly inducing apoptosis, can still affect key apoptotic regulators. For instance, increased Bcl-2 mRNA could be part of a broader cellular stress response or signaling triggered by MTsSPc. Additionally, the elevated mRNA levels of pro-apoptotic genes such as Caspase 9 and Cytochrome c, together with Bcl-2, may indicate a cellular compensatory mechanism. The cell may be trying to balance pro- and anti-apoptotic factors in response to MTsSPc, without triggering apoptosis. It needs additional experiments to investigate the residual activity of MTsSPc and explore the potential involvement of alternative pathways for providing a clearer understanding of the underlying mechanisms of the observed discrepancies.

Programmed cell death is mediated by caspases. Apoptotic caspase function is predominantly to initiate and execute apoptosis, and categorized into initiator and effector caspases based on their sequential roles in the apoptosis process [62,63]. Initiator caspases, such as Caspase 8 and Caspase 9, function as proteolytic signal amplifiers to activate effector caspases [64,65]. In turn, effector caspases like Caspase 3, proteolytically cleave various cellular proteins at specific target sites, thereby promoting apoptosis [66]. Apoptotic caspases are initially synthesized as inactive precursors known as procaspases, which are enzymatically inactive zymogens [67]. When cells were stimulated with apoptosis signals, the protease effector domain of procaspases (caspase domain) is cleaved into large and small subunits to form complexes for enzymatic activity, thereby regulating apoptosis [68]. In the present study, we investigated the expression of apoptosis-related proteins in Caco-2 cells treated with rTsSPc using qPCR and Western blotting. Our results showed that rTsSPc significantly increased the mRNA and protein levels of Caspase 3, as well as its cleaved form (cl-Caspase 3). Although the protein levels of Caspase 9 and Caspase 8 had no significant change, their cleaved subunits (cl-Caspase 9 and cl-Caspase 8) were clearly up-regulated, indicating the activation of these apoptosis-related proteins. Additionally, rTsSPc cleaved Poly ADP-ribose polymerase (PARP), a downstream substrate of Caspase 3, further confirming Caspase 3 activation [67] The Bcl-2 protein family, which regulates mitochondrial outer membrane permeabilization and controls the release of Cytochrome c, also showed an evident increase in expression level [68]. Specifically, the pro-apoptotic protein Bax and Cytochrome c were upregulated, while the anti-apoptotic protein Bcl-2 was downregulated in rTsSPc-treated cells [69,70]. These findings demonstrated that rTsSPc activated the apoptosis signaling pathway by upregulating pro-apoptotic genes and downregulating anti-apoptotic genes, ultimately inducing gut epithelial cell apoptosis.

However, the rTsSPc increased the Bcl-2 mRNA level; it seemed to be contradictory with the overall conclusion of apoptosis induction. But the Bcl-2 protein expression was decreased. The phenomenon suggested that the Bcl-2 protein may be subject to enhanced degradation. This could be due to the activation of proteolytic pathways, such as the ubiquitin-proteasome system, which can selectively degrade anti-apoptotic proteins in response to pro-apoptotic signals. In addition, the rTsSPc-induced apoptosis might also activate multiple pro-apoptotic pathways that overrided the compensatory upregulation of Bcl-2 at the mRNA level. The final result is a decrease of Bcl-2 protein expression level, which is consistent with the overall apoptotic response observed in this study. To address this concern, additional time-course experiments are needed to better understand the temporal dynamics of Bcl-2 and other apoptosis-related genes [71,72].

It has been showed that rTsSPc disrupted intestinal epithelial barrier and down-regulated the TJs expression *in vitro* [17]. However, it remains unclear whether rTsSPc-induced apoptosis disrupts intestinal barrier. Hence, an apoptosis inhibitor, Z-VAD-FMK, was administered, and then intestinal barrier functions were measured in this study. When Caco-2 cells were pretreated with Z-VAD-FMK, and cell apoptosis was inhibited, the damage of rTsSPc-induced Caco-2 monolayer integrity was alleviated, as characterized by an increased TEER, decreased FD-4 flux and up-regulated protein expression levels of TJs including ZO-1, E-cad, Occludin and Claudin-1. The results indicated that the apoptosis inhibitor abolished and relieved the rTsSPc-disrupted gut epithelial integrity, suggesting that gut epithelial apoptosis was involved in destruction of gut epithelial barrier.

To further characterize the key molecules which participate in rTsSPc-regulating apoptosis and thereby disrupting intestinal epithelial barrier, we further analyzed the results of mass spectrometry of Caco-2 proteins interacted with rTsSPc, and found that rTsSPc was capable of binding to phosphoglycerate mutase family member 5 (PGAM5), which is a critical molecule involved in necrosis, apoptosis, and autophagy and participates in cell apoptosis [6,23,73,74]. Therefore, the IFT co-localization, GST pull-down and Co-IP were performed to verify the binding and interaction of rTsSPc and PGAM5 in this study. Our results revealed that rTsSPc was specifically bound with PGAM5 both in soluble cellular proteins and natural living cells. To investigate the role of PGAM5 in rTsSPc regulating intestinal epithelial barrier, specific siRNA and a PGAM5 inhibitor LFHP-1c were employed to block the expression of PGAM5 receptor; the apoptosis inhibitor Z-VAD-FMK was used to inhibit intestinal epithelial apoptosis. The results revealed that blockage of PGAM5 prevented rTsSPc disrupting the Caco-2 monolayer barrier integrity, as demonstrated by the increased TEER, reduced intestinal permeability, and up-regulated TJs expression. Intestinal pathogens (including *T. spiralis*) damaging intestinal epithelial barrier is the adaptation mechanism that is favorable for their invasion and survival in hosts [75]. The results of an *in vitro* larval invasive test indicated that rTsSPc promoted larval invasion of gut epithelium, whereas inhibition of PGAM5 receptors and apoptosis pathway impeded and suppressed the rTsSPc-mediated larval invasion, further testifying that gut epithelial PGAM5 receptors and cell apoptosis were involved in *T. spiralis* intrusion of gut mucosa. Since Z-VAD-FMK is a broad-spectrum caspase inhibitor and may modulate the non-apoptotic pathways (e.g., inflammation, pyroptosis), the targeted inhibitor (such as Z-DEVD-FMK specific for caspase-3) should be used in future experiments, which will precisely determine the specific modulation of caspase-3 to the apoptotic or non-apoptotic pathways.

However, this study still has some limitations. It is known that PGAM5 might also take part in multiple cell death pathways except apoptosis, such as pyroptosis, autophagy and necrotic death [73,76]. PGAM5 has been shown to regulate pyroptosis through the activation of the NLRP3 inflammasome. Given the inflammatory nature of gut barrier disruption, we hypothesized that PGAM5-mediated pyroptosis could contribute to the observed effects. In addition, PGAM5 is also involved in the regulation of autophagy, which can influence cell survival and gut barrier integrity [76,77]. Therefore, the potential interplay between PGAM5-regulated autophagy and gut barrier function should be investigated in future study. Besides, whether rTsSPc initiates and activates other cell death pathways via interacting with PGAM5 in *T. spiralis* infection is required to be investigated in the future study. In addition, our results indicated that rTsSPc induced cell apoptosis and disrupted the barrier functions of Caco-2 monolayer *in vitro*, but the *in vivo* role of TsSPc on inducing apoptosis and disrupting gut epithelial barrier in host is also necessary to be further studied. Additionally, the immune protection provided by vaccination of mice with rTsSPc against *T. spiralis* larval challenge also needs to be further evaluated [38]. Furthermore, the hierarchical relationship between TsSPc-PGAM5-apoptosis and previously reported MAPK/ERK1/2 activation was not clear. In this study, we focused on TsSPc’s role in disrupting intestinal epithelial barrier and promoting larval invasion through PGAM5-mediated apoptosis. Although previous research has implicated the MAPK/ERK1/2 pathway in TsSPc-induced barrier disruption, this study specifically explored the apoptotic pathway regulated by PGAM5, and the results revealed that PGAM5-mediated apoptosis significantly contributed to intestinal barrier disruption and larval invasion. The synergistic potential of inhibiting both the PGAM5 apoptotic pathway and the MAPK/ERK1/2 pathway needs to be further investigated.

In conclusion, the *T. spiralis*-secreted TsSPc bound and interacted with PGAM5 receptor in gut epithelial cells, activated the apoptosis pathway and induced apoptosis. This interaction reduced the TJs expression levels, thereby compromised the integrity of intestinal epithelial barrier *in vitro*. Additionally, the PGAM5 inhibitor LFHP-1c and the apoptosis inhibitor Z-VAD-FMK prevented the disruption of intestinal epithelial barrier caused by rTsSPc. The two inhibitors also suppressed *T. spiralis* larval invasion of Caco-2 monolayer. Our results suggested that apoptosis-induced epithelial cell death created the gaps among intestinal epithelial cells, facilitating larval invasion. As the epithelial cells undergo apoptosis, they shrunk and detached from gut epithelium, creating openings and disrupting gut epithelial integrity, as a result, the larvae exploited these gaps to penetrate intestinal mucosa. This study indicated that TsSPc participated in *T. spiralis* invasion of gut epithelium by specifically binding to PGAM5 and inducing apoptosis. These findings are valuable for elucidating the mechanism of *T. spiralis* invasion and provide new strategies for prevention and treatment of trichinellosis. TsSPc might be considered as a novel candidate vaccine target to block *T. spiralis* larval invasion and infection.

## Supporting information

S1 Table Raw data of Figs 1–6 in this study. (XLSX)

S2 Table IFT analysis of fluorescence intensity changes of TJs in various groups. (DOCX)

S3 Table qPCR results of mRNA level changes of TJs gene in various groups. (DOCX)

S4 Table Western blotting of the proteins expression changes of TJs in various groups. (DOCX)
