# Supplementary material for: A Trichinella spiralis serine protease triggers gut epithelial apoptosis and destroys the barrier integrity to mediate larval invasion
Source: PLoS Negl Trop Dis. 2025 Oct 30;19(10):e0013680. doi: 10.1371/journal.pntd.0013680 (PMC12591413; doi:10.1371/journal.pntd.0013680)
Supplement: S2 Table — (DOCX) [file pntd.0013680.s002.docx]

**S2 Table IFT analysis of fluorescence intensity changes of TJs in various groups*.**

| **Groups** | **ZO-1** | | |  | **E-cad** | | |  | **Occludin** | | |  | **Claudin-1** | | |
| --- | --- | --- | --- | --- | --- | --- | --- | --- | --- | --- | --- | --- | --- | --- | --- |
|  | **Fold** | ***t* value** | ***P* value** |  | **Fold** | ***t* value** | ***P* value** |  | **Fold** | ***t* value** | ***P* value** |  | **Fold** | ***t* value** | ***P* value** |
| rTsSPc vs PBS group | -0.38 | 7.98 | 0.0013 |  | -0.61 | 4.91 | 0.008 |  | -0.54 | 5.38 | 0.0058 |  | -0.53 | 9.94 | 0.0006 |
| Z-VAD-FMK+rTsSPc vs rTsSPc group | 2.28 | 7.78 | 0.0015 |  | 1.61 | 6.39 | 0.0031 |  | 1.93 | 6.97 | 0.0022 |  | 2.57 | 28.78 | < 0.0001 |
| rTsSPc vs NC-siRNA group | -0.63 | 6.39 | 0.0031 |  | -0.78 | 4.46 | 0.0112 |  | -0.37 | 9.079 | 0.0081 |  | -0.38 | 6.135 | 0.0036 |
| siPGAM5+rTsSPc vs rTsSPc group | 2.47 | 7.987 | 0.0013 |  | 2.3 | 3.324 | 0.0293 |  | 2.364 | 3.019 | 0.0392 |  | 2.83 | 3.328 | 0.0292 |
| LFHP-1c+rTsSPc vs rTsSPc group | 2.02 | 8.21 | 0.0003 |  | 2.11 | 10.81 | < 0.0001 |  | 1.52 | 5.684 | 0.002 |  | 1.78 | 3.778 | 0.0163 |
| LFHP-1c+Z-VAD-FMK+rTsSPc vs rTsSPc group | 1.93 | 7,503 | 0.0005 |  | 2.01 | 10.33 | < 0.0001 |  | 1.58 | 6.554 | 0.0011 |  | 1.85 | 4.136 | 0.0109 |

*The fold changes of TJs fluorescence intensity andstatistical results in various groups compared to their corresponding control group.
