# Supplementary material for: A Trichinella spiralis serine protease triggers gut epithelial apoptosis and destroys the barrier integrity to mediate larval invasion
Source: PLoS Negl Trop Dis. 2025 Oct 30;19(10):e0013680. doi: 10.1371/journal.pntd.0013680 (PMC12591413; doi:10.1371/journal.pntd.0013680)
Supplement: S3 Table — (DOCX) [file pntd.0013680.s003.docx]

**S3 Table qPCR results of mRNA level changes of TJs gene in various groups*.**

| **Groups** | **ZO-1** | | |  | **E-cad** | | |  | **Occludin** | | |  | **Claudin-1** | | |
| --- | --- | --- | --- | --- | --- | --- | --- | --- | --- | --- | --- | --- | --- | --- | --- |
|  | **Fold** | ***t* value** | ***P* value** |  | **Fold** | ***t* value** | ***P* value** |  | **Fold** | ***t* value** | ***P* value** |  | **Fold** | ***t* value** | ***P* value** |
| rTsSPc vs PBS group | -0.6 | 3.16 | 0.0342 |  | -0.89 | 10.75 | 0.0004 |  | -0.59 | 13.5 | 0.0002 |  | -0.51 | 9.63 | 0.0007 |
| Z-VAD-FMK+rTsSPc vs rTsSPc group | 0.54 | 3.49 | 0.025 |  | 0.16 | 15.46 | 0.0001 |  | 0.35 | 17.34 | < 0.0001 |  | 0.24 | 7.09 | 0.0021 |
| rTsSPc vs NC-siRNA group | -0.68 | 5.05 | 0.015 |  | -0.78 | 6.87 | 0.0023 |  | -0.79 | 7.052 | 0.0021 |  | -0.44 | 13.97 | 0.0002 |
| siPGAM5+rTsSPc vs rTsSPc group | 1.44 | 5.05 | 0.0072 |  | 1.71 | 12.65 | 0.0002 |  | 1.38 | 9.168 | 0.0008 |  | 3.08 | 10.19 | 0.0005 |
| LFHP-1c+rTsSPc vs rTsSPc group | 0.66 | 4.82 | 0.0053 |  | 0.21 | 5.17 | 0.0037 |  | 0.84 | 24.61 | < 0.0001 |  | 0.052 | 0.8464 | 0.63 |
| LFHP-1c+Z-VAD-FMK+rTsSPc vs rTsSPc group | 1.44 | 10.60 | < 0.0001 |  | 0.45 | 11.17 | < 0.0001 |  | 1.14 | 33.52 | < 0.0001 |  | 0.34 | 5.502 | 0.0027 |

*The fold change of TJs mRNA level and statistical results in various groups compared to their corresponding control group.
