# Supplementary material for: A Trichinella spiralis serine protease triggers gut epithelial apoptosis and destroys the barrier integrity to mediate larval invasion
Source: PLoS Negl Trop Dis. 2025 Oct 30;19(10):e0013680. doi: 10.1371/journal.pntd.0013680 (PMC12591413; doi:10.1371/journal.pntd.0013680)
Supplement: S4 Table — (DOCX) [file pntd.0013680.s004.docx]

**S4 Table Western blotting of The proteins expression changes of TJs in various groups*.**

| **Groups** | **ZO-1** | | |  | **E-cad** | | |  | **Occludin** | | |  | **Claudin-1** | | |
| --- | --- | --- | --- | --- | --- | --- | --- | --- | --- | --- | --- | --- | --- | --- | --- |
|  | **Fold** | ***t* value** | ***P* value** |  | **Fold** | ***t* value** | ***P* value** |  | **Fold** | ***t* value** | ***P* value** |  | **Fold** | ***t* value** | ***P* value** |
| rTsSPc vs PBS group | -0.23 | 13.54 | 0.0002 |  | -0.69 | 3.97 | 0.0166 |  | -0.89 | 3.66 | 0.0216 |  | -0.44 | 10.73 | 0.0226 |
| Z-VAD-FMK+rTsSPc vs rTsSPc group | 3.86 | 4.71 | 0.0093 |  | 0.92 | 4.5 | 0.0108 |  | 0.35 | 2.8 | 0.0489 |  | 1.62 | 3.61 | 0.0004 |
| rTsSPc vs NC-siRNA group | -0.44 | 11.53 | 0.0003 |  | -0.35 | 4.508 | 0.0108 |  | -0.53 | 6.786 | 0.0025 |  | -0.43 | 17.09 | < 0.0001 |
| siPGAM5+rTsSPc vs rTsSPc group | 0.89 | 3.111 | 0.0358 |  | 0.61 | 4.756 | 0.0089 |  | 1.02 | 3.716 | 0.0205 |  | 1.09 | 4.514 | 0.0107 |
| LFHP-1c+rTsSPc vs rTsSPc group | 2.12 | 3.086 | 0.0367 |  | 1.57 | 3.477 | 0.0254 |  | 2.09 | 3.91 | 0.0174 |  | 1.54 | 5.549 | 0.0052 |
| LFHP-1c+Z-VAD-FMK+rTsSPc vs rTsSPc group | 2.43 | 2.861 | 0.0459 |  | 1.81 | 3.427 | 0.0315 |  | 2.18 | 7.158 | 0.002 |  | 1.69 | 5.141 | 0.0068 |

* The fold changes of TJs proteins expression levels and statistical results in various groups compared to their corresponding control group.
